# Ultra-miniaturized structured-light projector for tissue profiling in chip-on-tip endoscopy

**DOI:** 10.1117/1.BIOS.3.4.045002

**Published:** 2026-11-11

**Authors:** Marie C. Odenthal, Florence Auer, Hans Zappe

**Affiliations:** University of Freiburg, Laboratory for Micro-Optics, Department of Microsystems Engineering – IMTEK, Freiburg, Germany

**Keywords:** structured illumination, diffractive optical element, pattern projection, chip-on-tip endoscopy, tissue profiling

## Abstract

**Significance:**

Optical technologies can be used to accelerate the process of cancer diagnostics. For colorectal cancers, for example, one of the crucial parameters in classifying colon polyps is their shape and size, both of which can be optically characterized with structured light illumination.

**Aim:**

We develop an ultra-miniaturized structured-light projector suitable for chip-on-tip architecture that projects a desired pattern onto an area of 5  cm×5  cm at a working distance of 3 cm facilitating one-shot surface evaluation of the tissue to be examined.

**Approach:**

The pattern projection is based on a diffractive optical element with pixel sizes down to 200 nm enabling high diffraction angles of up to 40 deg. The entire setup consisting of a vertical-cavity surface-emitting laser, a collimation lens and the diffractive optical element is miniaturized to fit into the space constraints of the chip-on-tip architecture.

**Results:**

The developed projector has a length of 3.9 mm and a diameter of 4.2 mm including a cover glass to protect the optical components. The projector generates a stripe pattern with a stripe spacing of 5 mm and the desired projection size.

**Conclusions:**

The results show the development of a compact projector for structured light illumination that fits into chip-on-tip endoscopes and allows one-shot structured-illumination of colon polyps.

Statement of Discovery This work utilizes diffractive optical elements as pattern generating diffusers to develop a miniaturized structured-light projector that fits into a chip-on-tip colonoscope and projects an infrared stripe pattern onto the tissue.

## 1 Introduction

As several studies show, the most common pathway of colorectal carcinogenesis goes via the development of adenomas that are initially harmless.^1^^,^^2^ Therefore, effective early detection screening and appropriate treatment is essential for reducing the incidence of colorectal cancer.^2^

To categorize colon polyps found during the screening, many classification systems are used in clinical practice. The polyps are classified based on several criteria including color, granularity, pit pattern, shape and size. The latter two are used as input parameters for the PARIS classification,^3^ from which the severeness of the suspicious tissue and the endoscopic resectability can be predicted.

Since polyp size is proven to correlate with the malignity of the lesion,^2^ the size determines the follow-up procedure after the discovery of the polyp.^4^ Polyps measuring 1 cm or longer are classified in the high-risk group for invasive growth of malignant cells into deeper tissue layers.^5^^,^^6^ Thus, the size also determines the treatment plan of the discovered lesions. As the mortality for minimally-invasive treatments is lower than for open surgery,^7^ endoscopic treatments are favoured. However, deep mucosa invasive polyps cannot be treated endoscopically and need to be resected by surgery.^8^

When characterized *in situ*, the three-dimensional size of polyps is usually estimated based on clinical experience and can only be verified after the excision. Several studies have shown that the clinicians tend to underestimate the size of a colon polyp, which distorts the accurate assessment of the treatment plan.^9^^–^^11^ Due to the strong barrel distortion of the endoscopic camera, an accurate estimation of the size without significant experience is very difficult to obtain.^10^^,^^12^

To provide a more objective basis for the shape and size estimation of colon polyps, other technologies can be added to the endoscope. Using a forceps as a mechanical comparison tool faces challenges as the forceps can not be opened large enough to compare its opening with polyps of larger diameters.^10^^,^^12^ Other work has shown the development of ruler snares^11^ or ruler needles,^13^ which are marked at regular intervals to compare the size of the polyp. The systems yield an improvement of the visual size estimation for small polyps <5  mm^11^ or an improvement of the classification into categories of <10 and >10  mm.^13^ However, especially for larger polyps, the size estimation still suffers from a low accuracy of less than 20%.^11^ Although the preferred method to compare the polyp size is a linear ruler,^14^ linear measurement tools can not display polyps extending across mucosal folds.^10^ As a result, since the addition of a measurement step into the clinical workflow is always time-consuming, clinicians generally stick to the long-established visual estimation of the polyp size.^13^

However, to avoid disrupting the clinical workflow, other technologies can be incorporated into the endoscope. Optical technologies are particularly well-suited for this purpose, as they operate without physical contact and are therefore less prone to damaging the tissue. One optical technology with considerable promise to assess the surface structure of the examined tissue is that of structured illumination. All subtypes of structured illumination are based on the distortion of a known target pattern by a shaped surface.^15^ Structured illumination relies on the same principle as stereo vision, in which depth resolution is achieved through two different views of the same scene; in structured illumination, however, one of the views is replaced by a pattern projection system.^15^ To achieve depth resolution, the camera and projector must therefore view the same scene from two different positions.^16^

Simple pattern projections such as lines,^17^^,^^18^ grids,^19^^,^^20^ and dots^15^ have already proven the applicability of a structured light system in surface profiling. The pattern is projected onto the surface and captured with a camera from a different angle or position. The distortion of the pattern is registered, and the shifts and distances are used to calculate the underlying surface shape.^21^

To enhance the reconstruction and resolution of the surface profiling, regular projection patterns can be replaced with more complex ones.^16^^,^^22^ Examples include the application of sinusoidal patterns,^22^^,^^23^ color coding^24^^,^^25^ or irregular grids based on multiple elements^26^^,^^27^ reaching a reconstruction error of less than 1%^27^ or a geometrical accuracy of 0.1 mm.^24^ Spatial frequency domain imaging (SFDI) uses a sinusoidal pattern and can also give information about the optical properties of the illuminated sample.^23^^,^^25^

However, the existing endoscopy systems for pattern projection tend to be physically large due to the complexity of the components. Often, the complex setup is located outside the endoscope, and the light pattern is guided through the endoscope channel via a lens stack^21^^,^^23^ or optical fibers.^15^^,^^28^ Usually, the probe either blocks the working channel of the endoscope or can be used as a clamp-on to the side of the endoscope leaving the working channel free for surgical instruments.^28^ Some works show the miniaturization of side-looking probes;^18^^,^^24^ however, the side-looking feature complicates the use in combination with an endoscopy system as the probe must protrude from the distal end of the endoscope, which blocks the view of the imaging optics.

For both the pattern generation methods via shadow mask^27^ and via diffractive optical element (DOE)^15^^,^^19^ there is no truly miniaturized system compatible with chip-on-tip endoscopy requirements. In chip-on-tip endoscopy, all electrical and optical components, such as image sensors, light sources and lenses, are integrated into the distal tip of the endoscope. This approach offers several advantages over fiber-based concepts including, for example, improved image quality and lower chromatic aberrations.^29^ However, to ensure sufficient flexibility of the distal tip of such an endoscope, the tip components must be assembled as compactly as possible. While there is no guideline stating a maximum length for chip-on-tip endoscopy, probes reaching up to a length of 10 mm have been demonstrated in chip-on-tip endoscopy.^30^ As a result, both the diameter and the length of all components in the distal tip need to be strongly miniaturized to ensure the compatibility with the chip-on-tip architecture.

To allow single-shot measurements of colon polyps, the projected pattern should span a larger area than the typical sizes of colon polyps. Considering spreading tumors and giant polyps, polyps with diameters of more than 2 cm can be found in the colon.^8^^,^^31^ The thus required projection angle, in connection with the limited system size, leaves the use of a DOE as pattern-generating diffuser in the projection unit as one of the only viable options. In this context, a DOE is a transparent phase mask consisting of multiple square pixels in different level heights^32^; when illuminated with a collimated coherent laser source, this phase mask generates a pre-designed fringe pattern through diffraction.

We present here the proof-of-principle for the miniaturization of a structured-light projector into the chip-on-tip size requirements. The miniaturized projector is integrated into the distal tip of a chip-on-tip endoscope to provide the endoscope with a size-detection feature in addition to the capability for standard imaging. As the pattern projection capability of DOE is very versatile^32^ and previous work has already shown the successful application of a pattern projection in tissue profiling,^15^^,^^17^^,^^19^ we show the miniaturization with a DOE designed for a simple arbitrary stripe pattern, which can be later adapted to the requirements of the application.

The projector uses near-infrared (NIR) light, which, on the one hand, relaxes the miniaturization process since a vertical-cavity surface-emitting laser (VCSEL) can be used for illumination. On the other hand, using this wavelength range also enables the simultaneous application of the pattern projection with the standard visible-wavelength imaging used for clinical inspection. This allows the clinician to carry out the routine workflow without interruption of a time-consuming application of the measurement method. The projector is tested on pig mucosa to verify the visibility of the pattern.

## 2 System Design

### 2.1 System Requirements

As explained above, for an optical system to be compatible with endoscopic chip-on-tip architecture, all components and the complete system assembly must be strongly miniaturized.^30^ As a result, we define the miniaturization target for the structured-light projection system here as a maximum diameter of 5.5 mm and a maximum length of 6 mm, which, together with the imaging optics, would already fill the entire available space in the distal tip of the endoscope, assuming an outer diameter of the endoscope tip of 13 mm. To enable the additional integration of a working channel (typical diameter approximately 3 mm) and fluidic channels, the diameter of the projector must be significantly smaller than 5.5 mm, namely, below 3.5 mm.

To enable the simultaneous detection of the NIR pattern and standard visible-wavelength white light imaging, an image sensor which incorporates infrared-detecting pixels is required. Therefore, the image sensor *OV2744* (OmniVision, USA) is used in the design of a prototype. The OV2744 has a pixel size of 1.4  μm in a 16:9 full HD format. For initial depictions of the projection pattern within the dimensions of a chip-on-tip endoscope, the imaging of the projected pattern is conducted with a proprietary objective lens with a mechanical diameter of d=1.6  mm, a mechanical length of 5.5 mm and a field of view (FOV) of 90 deg, optimized for a working distance of 30 mm. Therefore, the projection pattern is also specified for a working distance of 30 mm. In the NIR region, modulation transfer function (MTF) measurements based on a knife-edge setup have shown a resolution of approximately 0.35 mm for the selected working distance.

Figure 1(a) shows the overlap of the imaging FOV and the projected fringe pattern. The degree of overlap is calculated as the sum of the areas of the two circular segments defined by the common chord of the intersecting circles. The geometrical area of each segment is determined analytically using the radii and the distance between the centers of the imaging and the projection field of view (FOV), with the length of the chord derived using Heron’s formula.^33^ To simplify the system, both the imaging and the projection area are approximated as circles, although the current definition of the stripe projection is based on a square projection pattern. The value of intersection is normalized by the area of the projection FOV to yield the relative overlap of the imaging FOV and the projection FOV. For small projection angles, $\theta_{\mathrm{proj}}$, and sufficiently small distances between the optical axes of the projector and imager, $d_{c}$, the projection area lies within the FOV of the imaging optics, as shown in the figure, such that a single-shot fringe measurement would be possible. However, for fringe measurements, a certain angle or spacing between the optical axes of the projector and imager are needed,^16^ implying that $d_{c}$ should be as large as possible. Thus the system design requires determination of a compromise value for $d_{c}$, small enough to guarantee complete overlap between projected pattern and imager FOV, but large enough to enable fringe reconstruction.

**Fig. 1:**
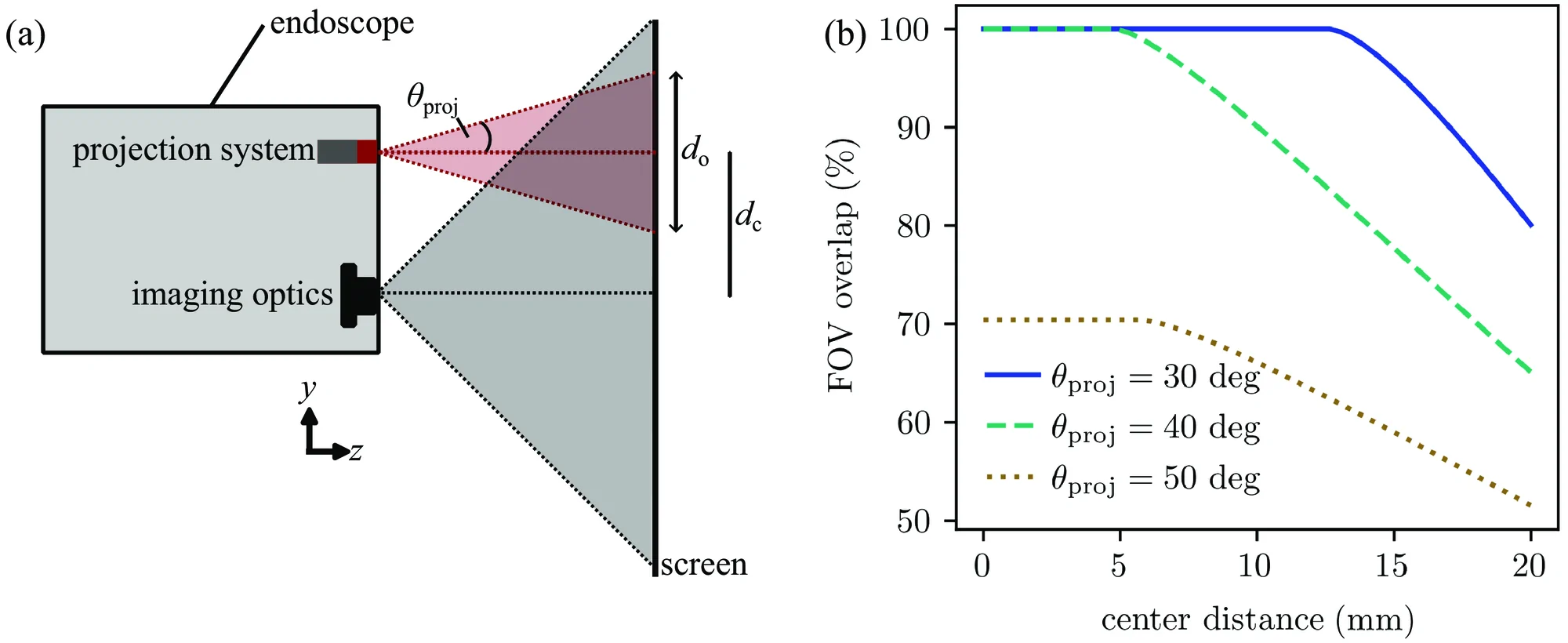
The center distance $d_{c}$ between the imaging optics and the projection system affects the recorded image of the distorted pattern. (a) Sketch of the field-of-view (FOV) of the imaging optics and an arbitrary pattern projected with an opening angle of $\theta_{\mathrm{proj}}$, where $d_{c}$ is the distance between the optical axes of the imaging optics and the projection system and $d_{o}$ is the resulting field overlap. (b) Field of view overlap as a function of $d_{c}$ when using different projection opening angles.

Because the size of the projected pattern must exceed the maximum size of colon polyps to cover the whole polyp in one shot, we thus specify a projection area of 50  mm×50  mm, corresponding to a projection angle $\theta_{\mathrm{proj}}$ of 40 deg. As is seen in the plot of Fig. 1(b), the maximum value of $d_{c}$ is thus about 5 mm to enable capturing the full projection FOV. Previous work has already presented the procedure for the design and fabrication of a suitable DOE for the required projection angle.^34^ However, as the particular DOE can be changed depending on the requirements, an adaptation of the projected pattern for a smaller pattern size or an increased spatial resolution is easily possible.

### 2.2 Diffractive Optical Element

The required structure of the DOE for the projection of the desired target pattern is computed using an iterative approach^35^^,^^36^ based on the Gerchberg-Saxton algorithm.^37^ The desired projection pattern is generated when the DOE is illuminated with a collimated laser source.

Figure 2(a) shows the dependence of the pixel size of the DOE and the maximum possible projection size at the working distance of 30 mm; these simulation results are generated using the mentioned Gerchberg-Saxton-based iterative algorithm. Because a DOE has a minimum periodicity given by the pixel size a, we can apply the grating equation for normal incidence and diffraction order m,

$$\sin\;\theta =\frac{m\lambda }{a},$$ (1)

to show that the maximum diffraction angle θ (and thus pattern size) varies as $\theta \propto a^{-1}$. Thus, the $x^{-1}$-fit to pattern size versus pixel size shown in the figure shows good agreement with the simulation results.

**Fig. 2:**
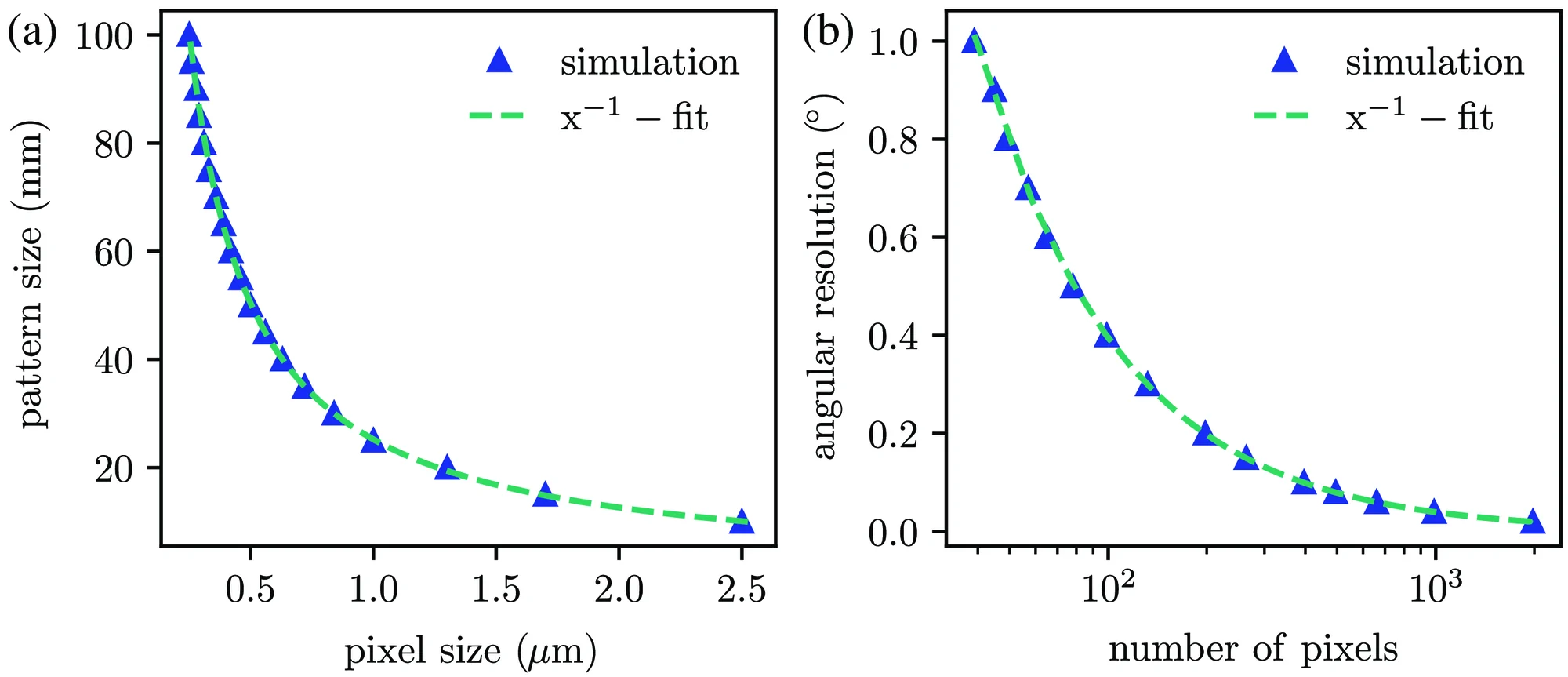
Effect of pixel size and number on the projected pattern. (a) Variation of the maximum projected pattern size in one dimension with pixel size. (b) Variation of the angular resolution as a function of the number of illuminated pixels in one dimension.

A common choice for the pixel size in other studies using pattern generating diffusers is 1  μm,^32^^,^^38^ which does not produce the required projection size and, at the working distance used here, can only illuminate an area of 25  mm×25  mm. To reach the defined projection area of 50  mm×50  mm, the pixel size must be smaller than 500 nm. For reasons explained elswhere,^34^ a pixel size of 200 nm is selected to generate the projection pattern with a safety margin.

Figure 2(b) relates the number of pixels per DOE to the achievable angular resolution of the projection pattern and clearly shows that angular resolution improves (decreases) as the number of pixels increases. Assuming an inverse relationship between the pixel number N and the angular resolution $(\Delta \theta {)}_{\min}$ of a simple grating structure,

$$(\Delta \theta {)}_{\min}=\frac{\lambda }{Na\;\cos\;\theta },$$ (2)

the data are again fit with a $x^{-1}$ relationship. The angular resolution influences the speckle-like structure of the projected pattern; small values of the angular resolution minimize visible discretization effects of individual intensity points and make the pattern appear more homogeneous and smooth. Due to a fabrication limitation, the DOE area is limited to 200  μm, which corresponds to 1000×1000  pixels with a pixel size of 200 nm and therefore an angular resolution of ∼0.04  deg.

## 3 Fabrication and Assembly

### 3.1 Diffractive Optical Element

To rapidly prototype DOEs for different target patterns, two-photon lithography (TPL) is used for the fabrication of the DOEs. TPL is a direct laser writing technology that volumetrically polymerizes an optical resin in the presence of two photons to generate truly three dimensional structures.

Figure 3 illustrates the process steps to print DOEs on glass substrates that consist of fused silica with a thickness of 0.7 mm and are coated with indium tin oxide (ITO). To facilitate the integration of the glass substrate into the tight chip-on-tip endoscope tip design, the glass substrate is diced into 2  mm×2  mm pieces. After the attachment of the glass piece to a transparent ground plate with transparent double-sided tape and the dispensing of optical resin, the actual two-photon lithography begins.

**Fig. 3:**
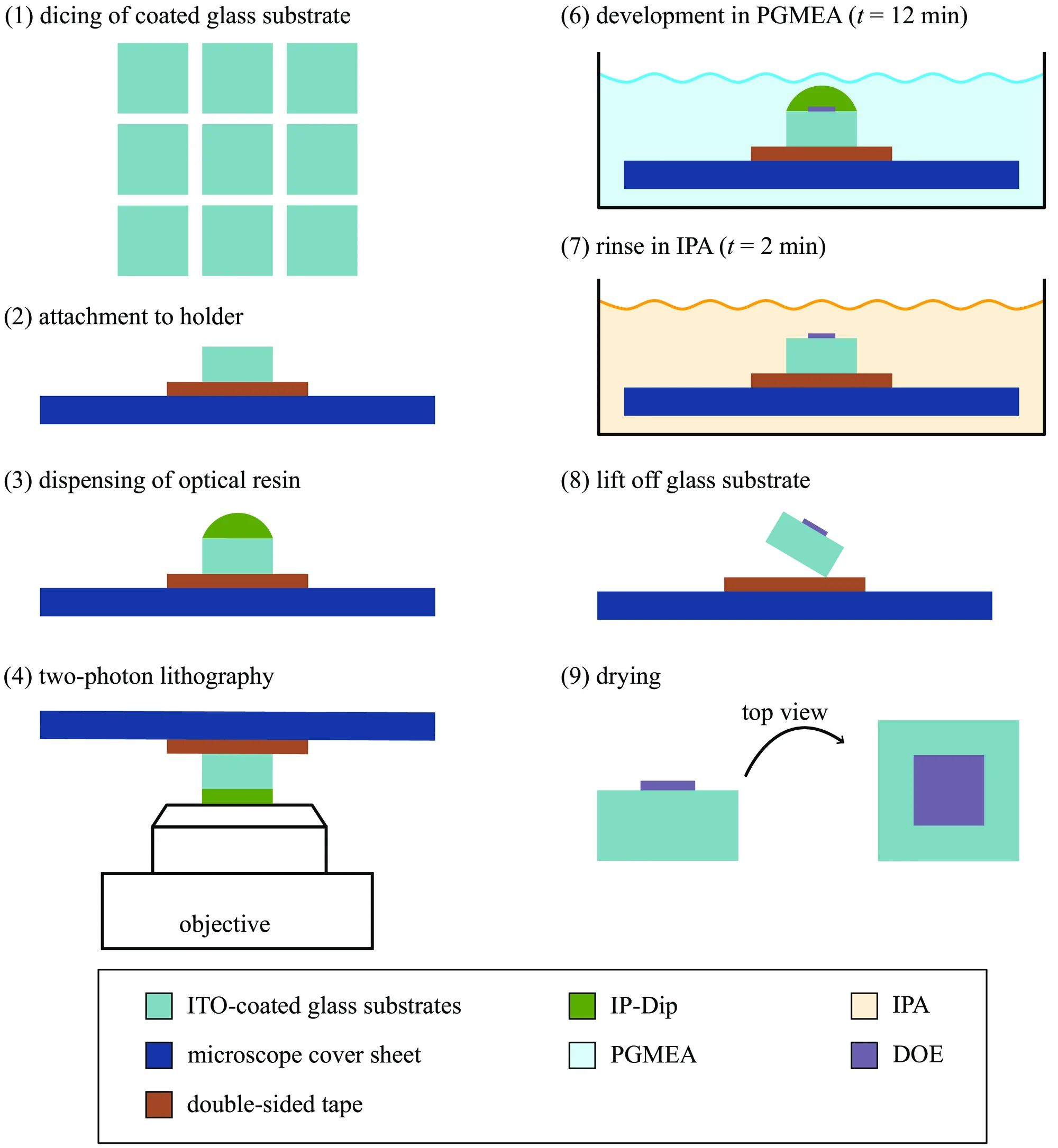
Rapid prototyping of a diffractive optical element via two-photon lithography.

For printing, the TPL system Photonics Professional GT+ (Nanoscribe, Germany) is used in combination with the optical resin IP-Dip (Nanoscribe, Germany) as recommended for the smallest feature sizes. Results of the printing process with an adaptation of the printing parameters and the printing method for optimized printing results has been described previously.^34^ The printing objective used here is a Plan-Apochromat 63x objective with NA=1.4 (Zeiss, Germany), which limits the writing field to 200  μm. To match the significantly larger beam diameter and direct the entire beam through the DOE, the DOE is repeatedly printed in so-called tiles. With a tiling of 7×7, the DOE is printed 49 times and the area covered is 1.4  mm×1.4  mm on a 2  mm×2  mm glass substrate, which amounts to a coverage of roughly 50% of the substrate area. Due to the enlargement of the DOE area, the alignment of the DOE with the collimated beam is less critical.

After printing, the exposed optical resin is developed in propylene glycol methyl ether acetate (PGMEA) for 12 min and excess resin is removed with isopropyl alcohol (IPA). During this process, the solvent already begins to dissolve the double-sided tape which allows the easy removal of the small glass piece with the DOE on top from the ground plate.

For subsequent steps, the DOE remains on the 2  mm×2  mm glass substrate and the glass itself is attached. On the one hand, the glass substrate ensures the mechanical stability of the DOE, because a deformation could strongly deteriorate the diffraction efficiency of the DOE. On the other hand, the slightly larger glass acts as a barrier. Because the DOE is smaller than the glass substrate, the adhesive can be applied to the surrounding glass margin without contaminating the diffractive optical area.

### 3.2 Projector

The illumination of the DOE is implemented with the single-mode VCSEL (VC850S-SMD, Roithner Lasertechnik, Austria) emitting a Gaussian beam with a divergence angle of 8 deg. The selected VCSEL has a center wavelength of 850 nm with a spectral bandwidth of 0.26 nm resulting in an estimated coherence length of 2.7 m, and an optical power of 0.5 mW at a bias current of 3.5 mA. Figure 4(a) shows an optical simulation conducted with Ansys Zemax OpticStudio (Ansys Inc., USA) using a wavelength of 850 nm. Based on the results of the optical simulation, an aspheric collimation lens made of glass (354,140, Thorlabs Inc., USA) is placed 0.8 mm from the light emitting facet to collimate the light. The collimated light is deflected using the diffraction orders m=1 and m=−1 of a blazed grating as placeholder for the DOE. As is the case for the final projector, the DOE is placed on a 0.7-mm-thick glass plate with a refractive index of $n_{\text{glass}}=1.59$, which further refracts the rays at the interface between the glass and air.

**Fig. 4:**
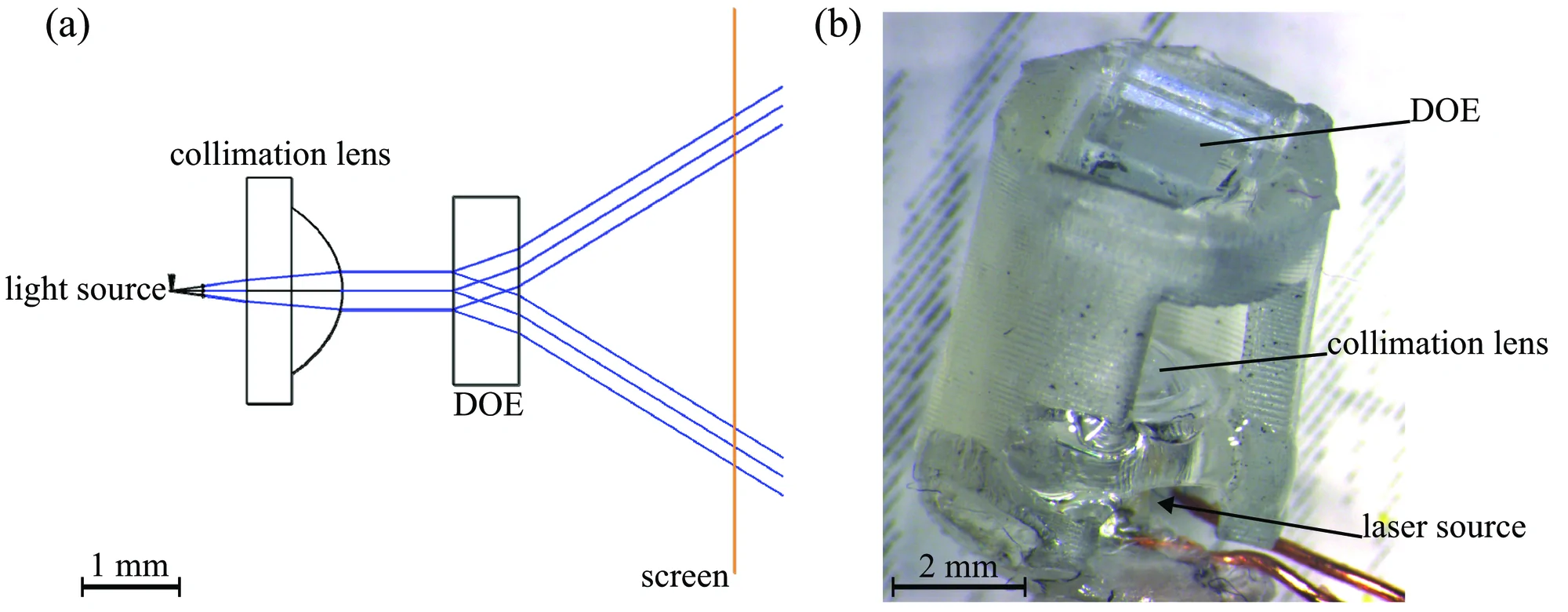
Alignment of the components of the miniaturized projection system. (a) Optical simulation of the beam collimation and the diffractive projection with diffraction modes m=1 and m=−1. (b) Assembly of the pattern projector consisting of the laser source, the collimation lens, and the DOE in a 3D-printed housing.

The support structures and the housing are 3D-printed with a clear resin to allow the visual alignment and inspection of the individual components. After electrical connection, the VCSEL is mounted to a round ground plate. The collimation lens, held in a 3D printed housing, is placed inside a hollow tube that is aligned with the light beam emitted by the VCSEL, whereas the collimation is monitored. The position of the VCSEL ground plate and the hollow tube of the collimation lens is fixed with UV-curable adhesive to enable a quick attachment and prevent misalignment.

The assembly of the pattern projector consisting of the VCSEL, the collimation lens and the DOE is shown in Fig. 4(b). As depicted in this image, the hollow tube that holds the collimation lens intentionally extends beyond the collimation lens to serve as a spacer to the next element. The depicted projector has a length of 5.8 mm and a diameter of 4.2 mm. According to the picture of the projector, the DOE is attached with a spacing of ∼2.5  mm above the collimation lens. As the DOE can also be mounted directly behind the collimation lens, a minimum projection system length of ∼3.9  mm can be achieved.

To show the integration of the pattern projector into a chip-on-tip endoscope, a simple endoscope tip model is built incorporating the key components, which are the pattern projector and imaging optics. Due to the proprietary nature of the imaging optics, a photograph of the endoscope tip model can not be given. However, Fig. 5 shows a cross-section of the design of the endoscope tip with these imaging optics, including an objective lens, light sources and a camera sensor, and the developed stripe projection unit, consisting of the VCSEL, a collimation lens and the DOE. In this model, the endoscope tip is designed with a diameter of 15 mm and the wall thickness of the projection unit housing is enlarged to provide more stability during measurements with the individual component. For the final integration of the projection unit in a chip-on-tip endoscope, the wall thickness can be reduced. Overall, the miniaturized size of the projection unit fits into the general chip-on-tip requirements. Due to the size of the largest individual component of 2  mm×2  mm, the overall size of the system can be reduced to this dimension and there are only slight adjustments of the housing necessary to fit into a fully equipped endoscope with a diameter of 13 mm.

**Fig. 5:**
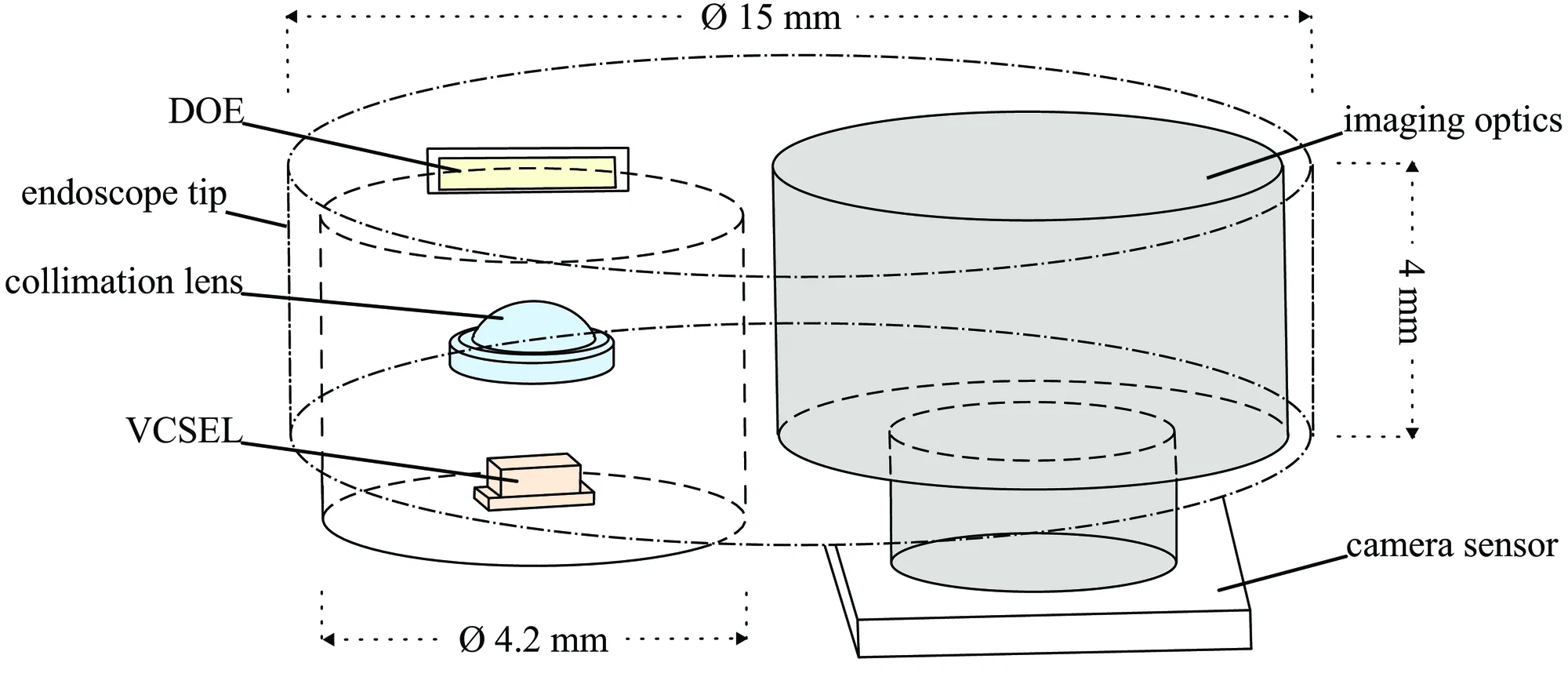
Cross-section of the distal tip of the endoscope including imaging optics and the projection system.

## 4 Pattern Projection

As the pattern projection capability of the DOE has already been examined elsewhere,^34^ the pattern projection is not further analyzed. For completeness, the projection results using the described endoscope tip system are summarized in Fig. 6. Figure 6(a) shows the simulated projection pattern generated using the Gerchberg-Saxton based algorithm to compute the DOE. As mentioned previously, the resulting pattern size enables one-shot measurements of colon polyps in various sizes up to 50 mm in diameter at 30 mm working distance. The projection of the endoscope tip model incorporating the pattern projector onto a planar screen is shown in Fig. 6(b). The image shows the distortion-free display of the stripe pattern. The corresponding image captured with the included optics [see Fig. 6(c)] reveals the offset between the center of the imaging and the projection. Compared with the desired pattern of 11 stripes and the simulation predicting 10 stripes, nine stripes can be recognized in the projection, with the brightness significantly decreasing toward the edges. Due to barrel distortion of the objective lens, the stripes no longer appear straight on a flat sample, which must be taken into account in calibration during subsequent analysis.

**Fig. 6:**
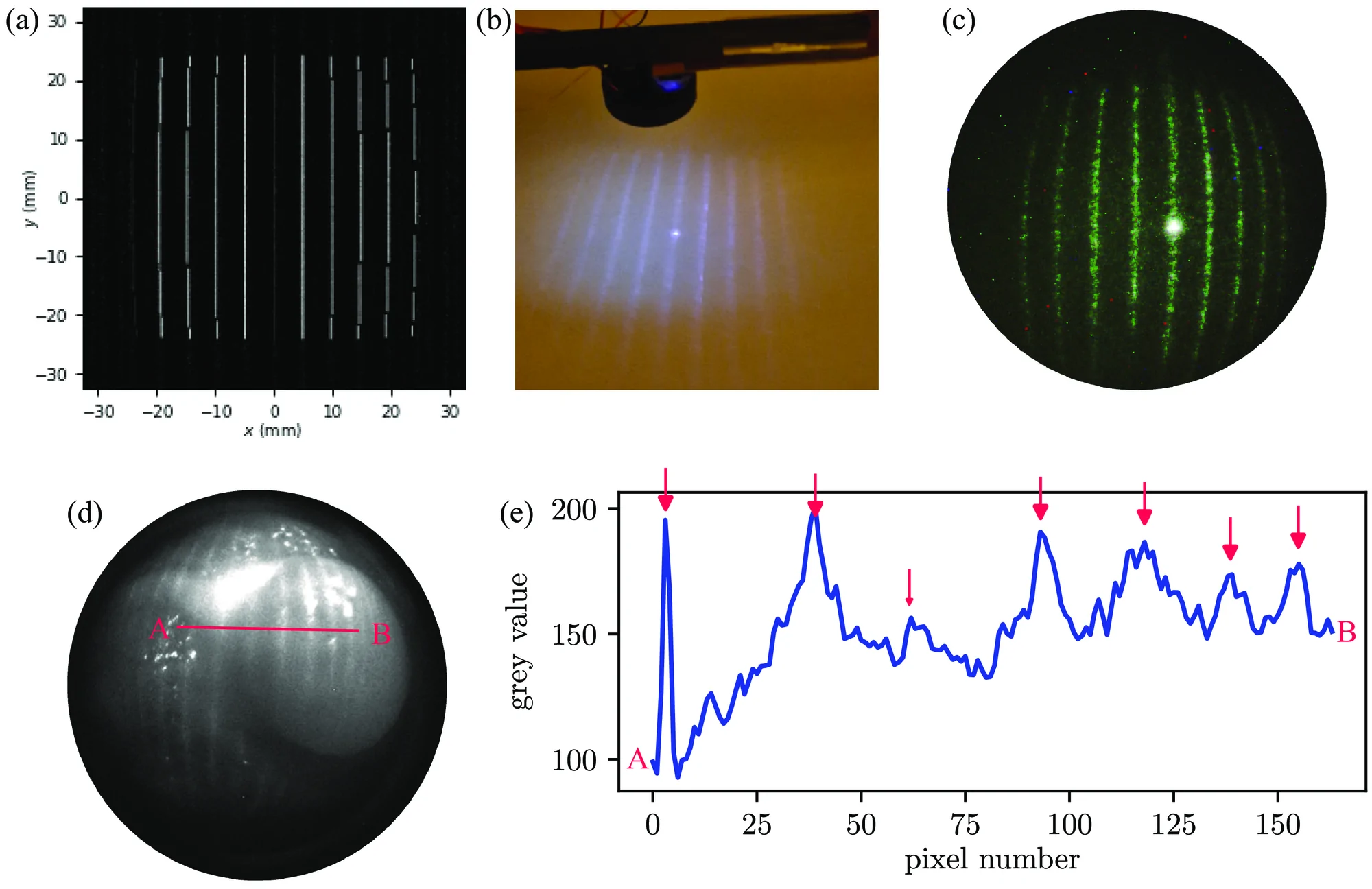
Pattern projection of a 50  mm×50  mm stripe pattern with a stripe spacing of 5 mm. (a) Simulation of the target pattern. (b) Measurement setup with the miniaturized projector held upside down, projecting the stripe pattern onto paper. (c) Projection pattern onto a flat paper screen captured with endoscope imaging optics. (d) Projection pattern onto polyp-like shaped pig mucosa captured with endoscope imaging optics. (e) Line scan A-B over seven stripes; stripe positions are labeled with red arrows.

Figure 6(c) shows the projection of stripes onto pig mucosa, with a surface shape similar to colon polyps, captured with the imaging optics integrated in the endoscope tip. The projected stripes are still visible as stripes, verified with the line scan shown in Fig. 6(d). The arrows indicate the position of the seven stripes included in the line scan, with the third stripe being hardly visible because of shadowing effects due to the shape of the mucosa.

## 5 Conclusion

We have presented a miniaturized stripe projection system compatible with chip-on-tip architecture requirements. The projection pattern meets the demands for the desired projection angle and enables one-shot measurements with a projection size of 50  mm×50  mm at a working distance of 30 mm. The near-infrared stripes are demonstrated to be clearly visible on pig mucosa allowing the inspection of biological tissue simultaneously with standard visual inspection by the clinician. Because the application of structured illumination for tissue profiling is well known, the developed system can be integrated into chip-on-tip endoscopes to give additional size measurements during the examination procedure. The DOE can be adapted to project arbitrary intensity patterns, thereby easing reconstruction or enhancing resolution, depending on the application envisaged.

Limitations arise from the projector design using an infrared laser source, as NIR light penetrates deeper into the biological tissue.^39^ On the one hand, this could broaden the application to gain information about deeper tissue layers, as the presentation of the pattern on different surfaces depends on the optical properties of the material.^23^ On the other hand, the pattern itself can suffer from blurring effects, as the NIR scattering inside the tissue may increase.

## Data Availability

The data that support the findings of this article are available from the corresponding author at marie.odenthal@imtek.uni-freiburg.de upon reasonable request.
